# Surgical Approaches to First Branchial Cleft Anomaly Excision: A Case Series

**DOI:** 10.1155/2016/3902974

**Published:** 2016-02-29

**Authors:** Lourdes Quintanilla-Dieck, Frank Virgin, Chistopher Wootten, Steven Goudy, Edward Penn

**Affiliations:** ^1^Department of Pediatric Otolaryngology Head & Neck Surgery, Oregon Health & Science University, Portland, OR 97239, USA; ^2^Department of Pediatric Otolaryngology Head & Neck Surgery, Vanderbilt University, Nashville, TN 37232, USA; ^3^Department of Pediatric Otolaryngology Head & Neck Surgery, Emory University, Atlanta, GA 30322, USA

## Abstract

*Objectives*. First branchial cleft anomalies (BCAs) constitute a rare entity with variable clinical presentations and anatomic findings. Given the high rate of recurrence with incomplete excision, identification of the entire tract during surgical treatment is of paramount importance. The objectives of this paper were to present five anatomic variations of first BCAs and describe the presentation, evaluation, and surgical approach to each one.* Methods*. A retrospective case review and literature review were performed. We describe patient characteristics, presentation, evaluation, and surgical approach of five patients with first BCAs.* Results*. Age at definitive surgical treatment ranged from 8 months to 7 years. Various clinical presentations were encountered, some of which were atypical for first BCAs. All had preoperative imaging demonstrating the tract. Four surgical approaches required a superficial parotidectomy with identification of the facial nerve, one of which revealed an aberrant facial nerve. In one case the tract was found to travel into the angle of the mandible, terminating as a mandibular cyst. This required* en bloc* excision that included the lateral cortex of the mandible.* Conclusions*. First BCAs have variable presentations. Complete surgical excision can be challenging. Therefore, careful preoperative planning and the recognition of atypical variants during surgery are essential.

## 1. Introduction

First branchial cleft anomalies (BCAs) are a rare finding in the head and neck. The incidence is estimated to be about one per million population/year [1]. Within the diagnosis of branchial cleft anomalies, first BCAs usually account for less than 8% but have been reported up to 21.4% in one series [1–3]. The malformations are described as distributed in the lateral neck below the external auditory canal (EAC), above the hyoid bone, anterior to the sternocleidomastoid muscle, and posterior to the submandibular angle [1].

There are four main theories of origin of BCAs, including incomplete obliteration of branchial mucosa, persistence of vestiges of the precervical sinus, thymopharyngeal ductal origin, and cystic lymph node origin [1]. In 1832, Ascherson first coined the term branchial cyst. He suggested that these malformations arise from incomplete obliteration of branchial cleft mucosa, which remains dormant until stimulated to grow later in life which results in cyst formation [1, 2]. Another theory postulates that brachial fistulas are vestiges of the cervical sinus, rather than of the pharyngeal clefts or pouches. Wenglowski et al. suggested that cystic degeneration of cervical lymph nodes was the mechanism of formation of lateral cervical cysts [1]. He also suggested that incomplete obliteration of the thymopharyngeal duct resulted in a lateral cervical cyst. Bhaskar and Benrier suggested that cystic alteration of cervical lymph node is stimulated by entrapped epithelium, where three possible sources are branchial cleft, pharyngeal pouch, and parotid gland.

A classification system proposed by Work in 1972 is as follows: type I lesions are anomalies that lie superficial to the facial nerve in close proximity to the ear, while type II lesions communicate with the EAC or tympanic membrane, often lying medial to the facial nerve [3]. The principles of management for these anomalies include early diagnosis, control of infection, and complete excision with facial nerve preservation.

## 2. Methods

Five cases of first branchial cleft anomaly treated surgically at our institution were chosen due to their representative nature of different clinical presentations, anatomical findings, and surgical approaches. A retrospective case review and literature review were performed.

**Figure 1:**
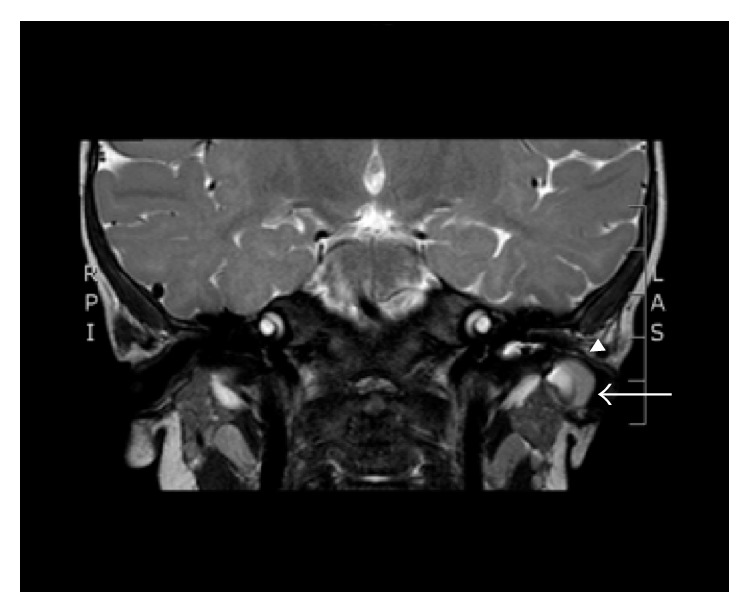
Case  1. MRI, coronal cut in T2 sequence, showing location of mass lateral to left parotid (arrow) and exerting mass effect towards the left EAC (arrowhead).

*Case  1*. An 11-month-old male initially presented with enlargement of the skin around the left EAC, causing occlusion of the canal. On examination, he was found to have a soft, ballotable area adjacent to the lateral EAC in the posteroinferior conchal bowl. He also had a small punctum in the lateral EAC floor just medial to the conchal bowl. He underwent a temporal bone MRI with and without contrast that showed a 1-centimeter lesion projecting inferiorly into the EAC, closely associated with the superoposterior parotid gland (see Figure 1). The lesion had slight rim enhancement on T1 sequence. The patient underwent surgical treatment. Using a lacrimal duct probe the tract was cannulated. The tract ended superficially, travelling adjacent to the EAC and not extending deeply into the parotid gland. Decision was made to excise the tract without need for a parotidectomy. An elliptical incision was made around the puncta and followed deeply with blunt dissection so as to not interrupt the tract. Fascial attachments were divided either bluntly or after confirming transillumination through the tissue. The borders of dissection were the cartilaginous ear canal posteriorly and skin and bony canal medially. The facial nerve monitor and Prass probe were used consistently throughout the case, never stimulating the facial nerve. The cyst tract, part of which was cartilaginous, was removed in its entirety. There were no complications and the patient made an uneventful recovery with no signs of recurrence at 18 months after surgery. 

**Figure 2:**
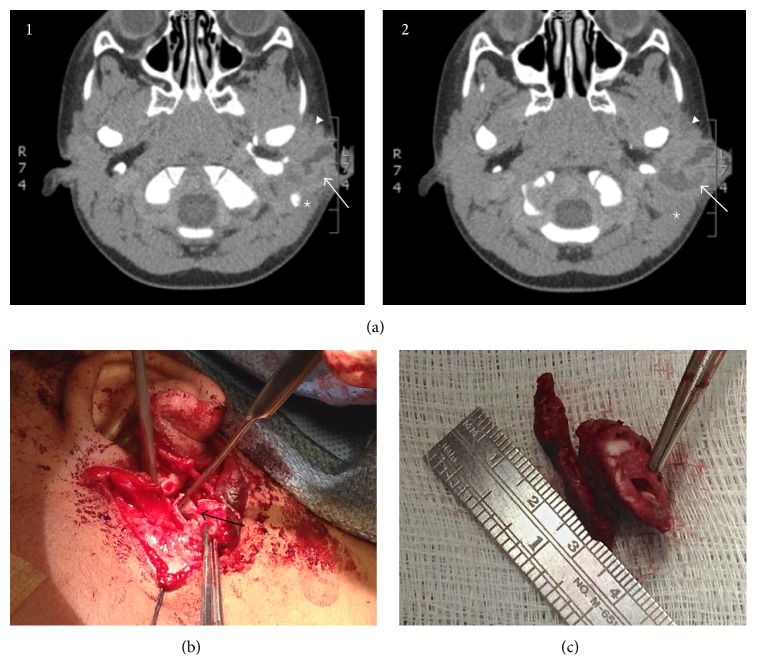
(a)(1-2) Case  2. Axial CT scan of the neck with contrast demonstrating a left rim-enhancing fluid collection (arrow) centered between the left parotid gland (arrowhead) and sternocleidomastoid muscle (star), posteroinferior to the EAC. (b) Duplicated EAC (*arrow*) containing skin and cartilage adnexae. (c) Specimen after removal. The cartilaginous duplication of the EAC along with its tract is shown.

*Case  2*. A 24-month-old female presented with a history of two episodes of left neck swelling, with drainage through a small infratragal pit as well as from the postauricular skin. A computed tomography (CT) scan of the temporal bones with contrast demonstrated a 2 × 2 cm abscess between the posterior parotid gland and the sternocleidomastoid muscle, just posteroinferior to the left EAC (Figure 2(a)). The fluid collection had thick rim enhancement and tapered as it extended toward the lateral aspect of the EAC up to the skin surface, narrowing the EAC. She was presumed to have a first BCA and was taken to surgery. The planned incision started along the pretragal crease and extended down and around the lobule, up along the postauricular crease, forming an ellipse around the skin where she had previously drained. A lacrimal probe was inserted deep into the inferolateral EAC puncta. A superficial parotidectomy was performed, where the tragal cartilage was found to be significantly enlarged, with medial extension. Dissection reached the tympanomastoid suture line but the nerve was still not visualized. At this point, the entire cyst tract could be visualized and the facial nerve was deemed to be within the deeper tissues. Therefore, the tract was removed starting with an elliptical excision around the puncta. Blunt dissection was carried out around the tract, travelling deeply and establishing an adequate plane around it. The probe was kept within the tract to assist with this dissection. The whole tract was separate from the normal EAC and deemed to be in fact a duplicated EAC (Figures 2(b) and 2(c)). After removal of the duplicated EAC and its tract including the postauricular fistula, the facial nerve was identified using the Prass probe. The facial nerve was located medial, inferior, and deep to the tympanomastoid suture line, medial to the elongated tragal pointer landmark. 

**Figure 3:**
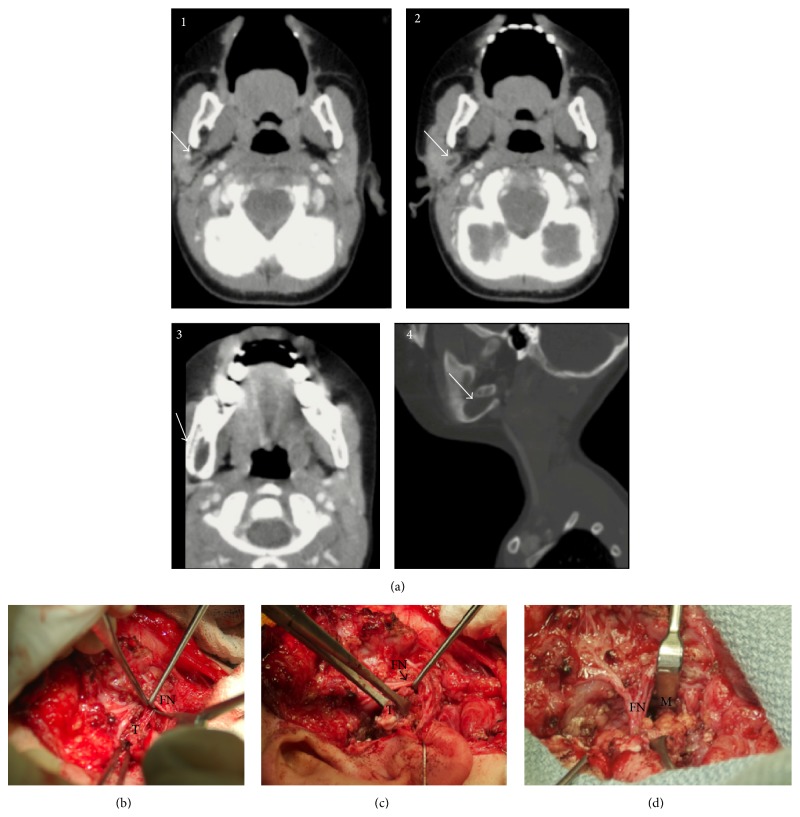
Case  3. (a)(1–4) Preoperative CT scans. White arrow depicts the right-sided lesion. (a)(1-2) Representative axial images revealing a well-defined right neck cyst (arrow) located posterior and deep to the parotid gland, with no clear connection to the skin. (a)(3-4) The extent of the right mandibular defect containing the cyst (arrow). (b) Identification of the tract extending deep to the facial nerve. In the inferior quadrant of the picture, a forcep is stenting the tract open as the facial nerve is being retracted. (FN: facial nerve; T: BCA tract). (c) Isolated tract after separating it from the facial nerve. A clamp is on the tract while the facial nerve is being retracted. (FN: facial nerve; T: BCA tract). (d) Tract extending medially entering the angle of the mandible. A clamp is on the tract (FN: facial nerve; M: mandible).

*Case  3*. An 11-month-old male presented with history of a preauricular pit excision. The report from that procedure stated that the pit was associated with a sac that “extended deeper than expected” but was excised completely. Two years later, he presented with swelling in his lateral EAC, leading to repeat surgery where a ruptured cyst was encountered. The cyst was followed through an endaural incision, extending deep to the parotid and toward the mastoid tip. It was not fully excised and left open with packing. After removal of the packing two weeks later, he was noted to have an abscess and a large red polyp in the EAC introitus. He underwent incision and drainage of the abscess, prompting referral to our hospital. A CT scan of the neck with contrast revealed a defect in the angle of the mandible associated with multiple cystic expansions of varying sizes. The wall of the larger cyst abutted the cystic encapsulation of an interrupted molar but did not involve the tooth (Figure 3(a)). The foramen of the inferior alveolar nerve was enlarged.

The patient was taken to surgery for definitive excision. He underwent a superficial parotidectomy, using a modified Blair incision that encompassed the pit identified in the EAC introitus. The facial nerve was identified and preserved. The fistulous tract was identified during the dissection along the tragal cartilage, lateral to the tragal pointer. It was dissected and traced anteroinferiorly where it travelled deep to the facial nerve (Figures 3(b) and 3(c)), towards the posterior aspect of the mandibular ramus and angle (Figure 3(d)). An exophytic bony growth encased the large cyst seen on imaging. Complete cyst removal required limited resection of the posterolateral aspect of the inferior half of the ramus and angle, enough to resect the entire cyst wall. A sufficient amount of anterior ramus and angle were left intact, and the strength of the mandible was not compromised. The facial nerve was protected throughout the procedure. The patient recovered well with postoperative course complicated by a temporary marginal mandibular weakness. He had no further recurrence of disease. 

**Figure 4:**
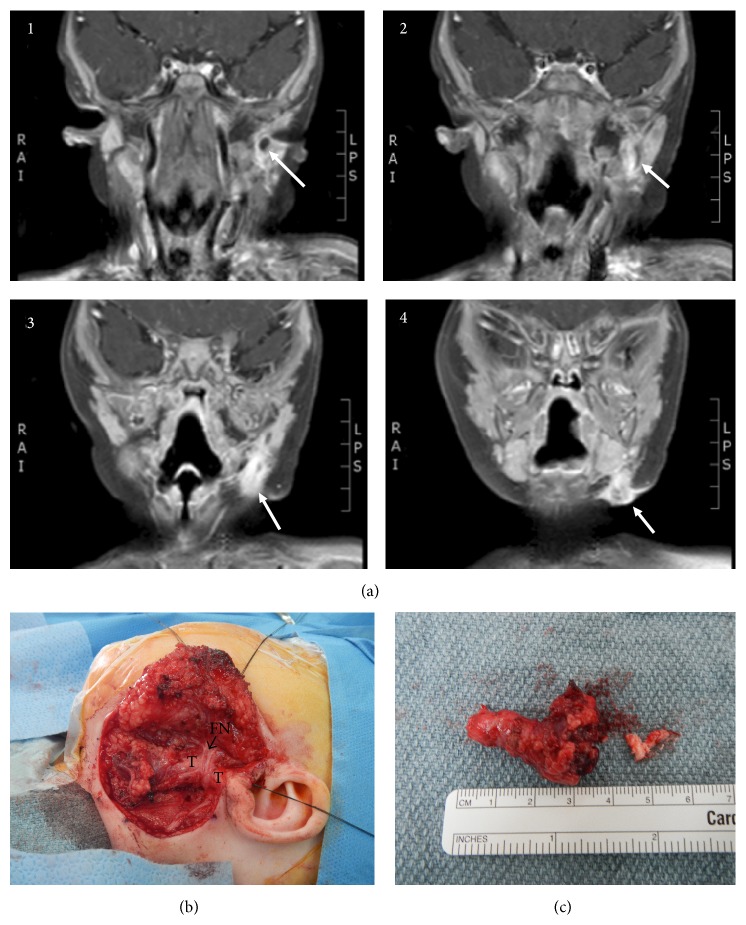
Case  4. (a)(1–4) MRI with and without contrast, T1-weighted images in coronal cuts, showing tract (arrow) travelling from adjacent to left EAC superiorly, down towards left submandibular fossa, posterior to the submandibular gland, and ending at the skin inferiorly. (b) Facial nerve identified and protected during surgery. The tract is seen passing deep to the nerve (FN: facial nerve; M: mandible). (c) Specimen after removal, consistent with a duplication of the EAC, containing cartilage (separate piece dissected off main specimen after excision).

*Case  4*. A 5-month-old male presented with a 3-day history of left submandibular erythema and swelling. His mother reported the presence of a pit in this area, present from birth. Needle aspiration was performed, revealing a small quantity of purulent fluid. The infection was treated with antibiotics and the swelling improved. At 8 months of age, definitive surgical treatment was undertaken. A preoperative MRI of the neck with/without contrast revealed a cystic enhancing structure in the left submandibular region, extending posterior to the mandible and superiorly to the EAC, and measuring 1 cm in greatest diameter along its tract (Figure 4(a)(1–4)). A modified Blair incision was made and dissection taken down to parotid fascia. The anterior border of the SCM and posterior digastric muscle were identified and the facial nerve was identified and followed anteriorly to the pes anserinus (Figure 4(b)). The cyst was identified in the left submandibular fossa, dissected, and separated from surrounding tissues. Its tract was followed superiorly, travelling medial to the inferior branch and main trunk of the facial nerve. The tract was carefully separated from the facial nerve and followed to the superior aspect of the EAC, where it ended as a blind pouch with no EAC defect. Given its close relationship to the normal EAC, a small portion of the cartilaginous EAC was removed along with the specimen (Figure 4(c)). The patient recovered well with no complications or recurrence. 

**Figure 5:**
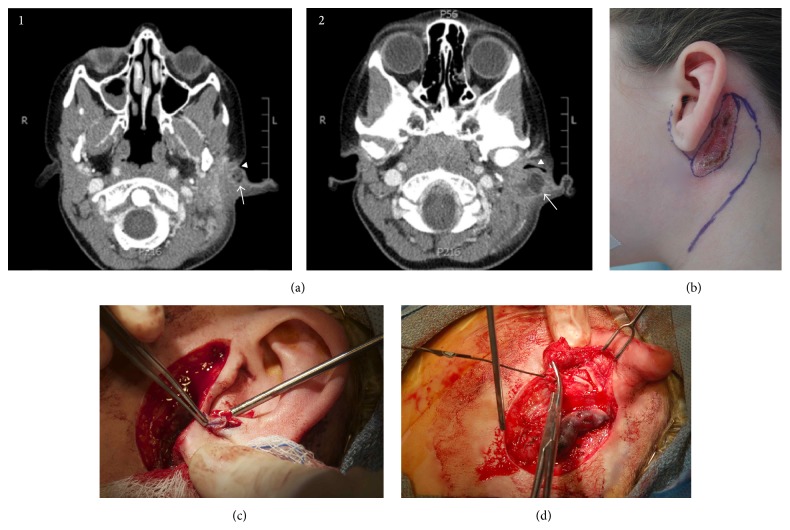
Case  5. (a)(1-2) CT scan with contrast, axial cuts demonstrating a left rim-enhancing postauricular abscess (arrow) adjacent to the conchal bowl (where puncta were identified at time of surgery), causing EAC stenosis (arrowhead). (b) Modified Blair incision incorporating postauricular granulation tissue (site of prior I&D). The limb of the marked incision extending down along a neck crease was marked as a possibility but not incised during the surgery, since it was unnecessary for visualization of the digastric and SCM muscles. (c) After identifying the facial nerve, an elliptical incision was carried out around the antitragal pit and dissected down. (d) Both the gentian violet within the tract and the lacrimal probe were used throughout the case to identify the tract. After initial dissection, the ellipsed skin around the pit and the contiguous tract was passed deep to the auricle and brought out through the postauricular incision keeping the entire tract intact.

*Case  5*. A 7-year-old female with a history of a left intermittently draining preauricular pit and a narrowed left EAC presented with foul-smelling drainage from the pit, left otalgia, and swelling with erythema of the postauricular skin. Imaging with a CT scan of the temporal bones with contrast was performed, revealing a 1.7 × 1.8 cm complex heterogeneous fluid collection located posterior to the conchal bowl and adjacent to the EAC, suggestive of an infected first BCA (Figure 5(a)(1-2)). She was treated with oral followed by intravenous antibiotics but ultimately required an incision and drainage (I&D) of the abscess. During the procedure, dermoid material was expressed from the pit; an incision was made in the postauricular skin and 1.5 mL of purulent drainage was obtained. She was treated with clindamycin after a culture grew mixed Gram-positive bacteria without a predominant organism. She required a repeat I&D several weeks later where a culture was positive for* Actinomyces*. She was seen by the Pediatric Infectious Disease service and required multiple courses of penicillin and trimethoprim-sulfamethoxazole due to recurrent swelling and pain.

Following resolution of an acute infection, an MRI of the neck with and without contrast was obtained. This demonstrated a minimal decrease in the size of the cyst when compared to the prior CT scan. She was taken to surgery for definitive treatment. A lacrimal probe was inserted into the puncta at the lateral edge of the conchal bowl, and gentian violet mixed with bacitracin was injected into the lumen of the tract for staining. During this injection, the dye was noted to extrude from the postauricular granulation tissue where the prior I&D had been performed. A curvilinear incision was traced from the pretragal crease, around the lobule, then continuing postauricularly in elliptical fashion around the scar and granulation tissue (a 4 × 2 cm area) (Figure 5(b)). A superficial parotidectomy was performed and the facial nerve was identified in its normal location. An ellipse was made around the auricular pit then dissected bluntly from surrounding tissues. The dye aided in identification of the lumen (Figure 5(c)). The tract was dissected deeply until it was connected to the postauricular fistula and scar tissue. These were kept intact and excised as one specimen (Figure 5(d)). During this dissection, the great auricular nerve was encountered and required sacrifice in order to keep the specimen intact. The tract was noted to be adherent to the conchal bowl and once resected, it resembled a duplicated EAC. Closure was performed in multilayer fashion with extensive undermining to achieve primary closure. Adequate auricular positioning was achieved during closure.

## 3. Discussion

First BCAs can appear at any age but are a relatively rare cause of congenital anomalies in children. Age of presentation varies and in one report it was 19 years of age [1]. The patients in our series presented with symptoms at an age as early as 5 months and underwent definitive surgical management as young as 8 months of age. They presented with either EAC stenosis or signs and symptoms of an acute infection. Other presenting signs and symptoms in the literature include primary otologic complaints such as otorrhea, otitis media, or cholesteatoma [3]. In spite of their rarity, it is important to keep this possible diagnosis in mind in any case of swelling or infection around the external ear. Misdiagnosis can lead to inadequate treatment and high risk of recurrence. Children with first BCAs have a higher incidence of undergoing initial incision and drainage, making the definitive surgical treatment more challenging [3].

First BCAs have been classified by Work into two different types, on the basis of clinical and histopathologic features. Type I anomalies are ectodermal and present as a cystic mass, with squamous epithelium but no skin adnexa or cartilage remnants. These occur superficial to the facial nerve, adjacent to the pinna, and often extending into the postauricular crease. Type II anomalies can present as cysts, sinuses, or fistula tracts and are of mixed ectodermal and mesodermal origin. On histology, they have squamous epithelium with skin adnexa or cartilage. They may pass through the parotid gland either superficial or deep to the facial nerve, and the tract may either end in the cartilaginous external auditory canal (possibly communicating with the tympanic membrane) or extend to the face or upper neck [1–3]. In our case series, 1/5 patients was classified as Work Type I first BCA, and 4/5 were Work Type II with skin adnexa with or without cartilage identified in the specimen.

Imaging studies can help delineate the anatomy of each branchial cleft anomaly, which can be especially helpful for determining the surgical approach. A computed tomography scan or magnetic resonance imaging will show a fluid-filled cyst with or without signs of infection and inflammation, depending on the timing of the scan. It can help delineate its size and anatomic relationships to important surrounding structures, such as the facial nerve and external auditory canal. In our series, two patients received a CT scan of the temporal bones or neck with contrast, two others underwent an MRI of the neck with or without contrast, and the fifth patient had both studies done at different time points. In all patients, imaging, regardless of the modality, was essential in preoperative planning and the operative approach to definitive treatment.

Many of the patients in our series required multiple drainage procedures prior to definitive surgical treatment. An absence of prior incision and drainage procedures is ideal at the time of definitive first BCA excision. Prior drainage or cyst rupture may lead to scar tissue and fistula formation. The reported recurrence rate can be as high as 14–22% after surgical excision when there is a history of prior infection or incomplete excision [2, 4]. In two of our cases a postauricular fistula developed at time of acute infection, requiring excision of this fistulous tract at time of complete resection. Ideally, definitive surgical excision should be done once any acute infection has resolved and the antibiotic course is finished. However, in cases of persistent and frequent symptoms such as swelling as pain, this time period may be shortened. If scar tissue or a fistula did form, these should be excised completely along with the tract and cyst.

Surgical excision is the definitive treatment for branchial anomalies, and the surgical plan needs to be tailored to each case. One of the keys to complete excision is keeping the tract, cyst, and any fistula or scar tissue intact. A lacrimal probe can be used throughout the case to ensure that the tract does not get disrupted and to determine the orientation of the tract. A dye such as methylene blue or gentian violet can be infused into the tract to help with identification throughout the case. One should also be prepared to remove parts of noncritical structures intimately related to the cyst or its tract, such as a section of the cartilaginous EAC or the posterior mandible.

Preservation of the facial nerve is another key issue in these surgeries. A common practice for first cleft anomalies given their location is to perform a superficial parotidectomy approach with facial nerve identification. However, the approach needs to be tailored to the characteristics of each specific case, which can vary widely. Only one patient in our series did not require identification of the facial nerve, given limited extent of the tract. However, there should be a low threshold to perform a superficial parotidectomy and identify the nerve. All cases used facial nerve monitoring, and even though the nerve was never stimulated unintentionally, the Prass probe proved to be helpful. One study by Bajaj et al. identified 15 patients with first BCA, and the facial nerve was identified in all cases prior to excision of the fistula [2]. In one of these patients, the tract was a fistula opening into the ear canal. In the same study, six cases had the sinus tract entirely deep to the facial nerve, while in eight patients the tract was superficial to the main trunk and branches of the nerve. In one case the fistula was superficial to the lower division and deep to the upper division of the facial trunk. This demonstrates the variability of relationship to the facial nerve and the importance of meticulous dissection in every case.

In any congenital anomaly, the facial nerve can be anomalous since its development begins after that of the first branchial arch derivatives. However, the classic landmarks to identify the facial nerve in a parotidectomy, such as the tragal pointer, tympanomastoid suture line, and the digastric/sternocleidomastoid muscles, can still be used in these procedures. For example, if the tragal cartilage is elongated deeper than usual, the nerve will still be deep to this cartilage. Facial nerve injury is a risk that must always be discussed with the family, especially in cases of prior infection or surgery. An older series reported facial nerve injury in 2/5 cases, while some newer series report anywhere from a 7–15% risk [2, 5, 6].

## 4. Conclusion

First BCAs are rare but must be kept in the differential diagnosis whenever a patient presents with swelling around the external ear or upper lateral neck. The surgical approach should be based on physical exam, imaging, and clinical course. It is important that the surgeon be aware of anatomic variants, additional tissue that may need to be excised (e.g., scar tissue, fistula, and adjacent non-critical structures), the need for safe identification of the facial nerve in almost all cases, and intraoperative tools that can assist with complete excision (e.g., lacrimal probe and dyes).
